# The vital importance of a web of prevention for effective biosafety and biosecurity in the twenty-first century

**DOI:** 10.1186/s42522-021-00049-4

**Published:** 2021-09-20

**Authors:** Tatyana A. Novossiolova, Simon Whitby, Malcolm Dando, Graham S. Pearson

**Affiliations:** 1 Research Fellow, Law Program, Center for the Study of Democracy, Sofia, Bulgaria; 2 Senior Lecturer, Peace Studies and International Development, University of Bradford, Bradford, UK; 3 Professor, Leverhulme Emeritus Fellow, Peace Studies and International Development, University of Bradford, Bradford, UK; 4 Visiting Professor (ret.) in International Security, Peace Studies and International Development, University of Bradford, Bradford, UK

**Keywords:** Biosecurity, Biosafety, Web of prevention, Biological and toxin weapons convention, One health, Health security, Deliberate disease

## Abstract

Biological threats are complex and multifaceted, as evidenced by the ongoing COVID-19 pandemic. Their effective prevention and countering require multiple lines of collaborative action and sustained cross-sectorial coordination. This paper reviews the conclusions of Graham Pearson’s 1997 *JAMA* article titled ‘The Complementary Role of Environmental and Security Biological Control Regimes in the 21st Century’, taking into account the international policy developments that have occurred over the past two decades. The paper underscores the utility of the concept of a ‘web of prevention’ for elucidating the need for continuous interaction between the international biosafety and international biosecurity regimes, in order to ensure that the life sciences are used only for peaceful purposes. The terms ‘biosafety’ and ‘biosecurity’ are used to denote the primary purpose of the two regimes: the international biosafety regime seeks to prevent the unintentional (accidental) release of pathogens and toxins, including naturally occurring disease, whereas the biosecurity regime seeks to prevent the deliberate release and misuse of pathogens and toxins. The paper concludes by recommending practical steps for strengthening the implementation of all elements of the web of prevention and upholding the norms against the hostile misuse of life sciences.

## Introduction

A 1997 article titled ‘The Complementary Role of Environmental and Security Biological Control Regimes in the 21^st^ Century’ that appeared in the *Journal of the American Medical Association (JAMA)* argued that:

“As we approach the 21st century, there is increased worldwide concern about disease, whether natural or deliberate, in humans, animals, and plants. There are 2 driving forces for multilateral biological control regimes: international/national security and environmental protection. With respect to deliberately caused disease, these seemingly disparate forces are mutually reinforcing as demonstrated by simultaneous moves to strengthen the Biological and Toxin Weapons Convention and the entry into force of the Convention on Biological Diversity. Future multilateral biological control regimes based on these developments will aid the security, prosperity, and health of the world community” [1].

More than two decades later, it is evident, not least from the ongoing COVID-19 pandemic that biological threats regardless of their origins constitute a major security concern. Biological threats are complex and multifaceted and hence, their effective prevention and countering require multiple lines of collaborative action and sustained cross-sectorial coordination. It is helpful to think of this required approach as an integrated and comprehensive web of prevention in which the efforts aimed at preventing the accidental release of biological agents or toxins, including naturally occurring disease and the efforts aimed to prevent the deliberate release of biological agents and toxins and the misuse of life sciences are complementary and reinforce each other. This paper reviews the conclusions of the 1997 *JAMA* article against the backdrop of the dynamic international security context, on the one hand, and the rapidly evolving life science landscape, on the other. In doing so, it underscores the utility of the concept of a ‘web of prevention’ for elucidating the need for continuous interaction between the international biosafety and international biosecurity regimes, in order to ensure that the life sciences are used only for peaceful purposes. The terms ‘biosafety’ and ‘biosecurity’ are used to denote the primary purpose of the two regimes: the international biosafety regime seeks to prevent the unintentional (accidental) release of pathogens and toxins, including naturally occurring disease, whereas the biosecurity regime seeks to prevent the deliberate release and misuse of pathogens and toxins. The paper examines the evolution of the spectrum of biological risks over the past two decades to account for the growing recognition of the complementarity of biosafety and biosecurity for preventing the accidental or deliberate misuse of the life sciences. It then introduces the concept of a ‘web of prevention’ to outline the key elements of the international biosafety and biosecurity regimes and highlights the need for the development of integrated national implementation approaches for the management of biological threats. The paper concludes by recommending steps to be taken for strengthening the implementation of all elements of the web of prevention and upholding the norms that the life sciences and related fields are used only for peaceful purposes and the benefit of mankind and the environment.

### The Spectrum of biological risks in the twentieth-first century

Disease outbreaks are classified as naturally occurring, accidental, and deliberate. Traditionally, each type of disease has been addressed by a different set of preventive measures: public health measures are used to prevent naturally occurring disease; laboratory biosafety measures are used to prevent disease as a result of industrial and laboratory accidents and/or negligence; and biosecurity, including disarmament measures are used to prevent deliberate disease. Yet determining the origins of a disease outbreak is far from straightforward and in certain cases, it may even prove to be impossible [2]. The ongoing debate on the origins of the SARS-CoV-2 virus is indicative in this regard. In March 2021, the World Health Organisation (WHO) published the report of a joint WHO-China study which was carried out in January–February 2021 as part of the international efforts to identify the source of the virus and how it was introduced in the human population [3]. According to the report findings, the introduction of the virus in the human population through an intermediate host is considered a likely to very likely pathway, whereas the introduction of the virus through a laboratory incident is considered an extremely unlikely pathway [3]. The report has been met with mixed reactions within scientific and policy circles, particularly as it has raised more questions than it has answered [4]. In a letter to the *Science* magazine, a group of prominent scientists have noted the need for an investigation that is “transparent, objective, data-driven, inclusive of broad expertise, subject to independent oversight, and responsibly managed to minimise the impact of conflicts of interest” [5]. This call was echoed by the US National Academies of Sciences, Engineering and Medicine (NASEM) in a recently issued statement [6]. The statement cautions against the negative effects of “misinformation, unsubstantiated claims, and personal attacks on scientists surrounding the different theories of how the virus emerged” noting that these are unacceptable and risk undermining the public’s trust in science and scientists [6].

Disease recognises no borders and the globalised systems of international travel, trade, and communication can turn a local event into a global crisis, as the 2004 SARS, 2014–2015 Ebola outbreaks, and the current COVID-19 pandemic have demonstrated. Antimicrobial resistance coupled with the impact of climate change on disease patterns and the re-emergence of vaccine-preventable diseases are symptomatic of the challenges that global public health faces [7]. The progress of biotechnology over the past few decades promises to make a significant contribution to addressing some of these issues, for example, through the generation of novel drugs and therapeutics. It also promises to advance sustainable development through the provision of products and technologies that can help reduce the environmental footprint of human activity, alleviate poverty, and protect biological diversity [8].

At the same time, there are concerns that the global diffusion of cutting-edge life science capabilities, such as genome-editing and synthetic biology, both in and outside traditional research environments (e.g. emergence of community laboratories and ‘do-it-yourself’ biology movement) increases the risk of accidental and deliberate misuse of life science knowledge and materials against humans, animals, or plants [9]. The advent of enabling life science advances, the possibility of acquiring biological agents and toxins via illicit online markets (e.g. Darknet), and the ever-increasing open-access pool of scientific knowledge constitute critical governance (and security) challenges that can hardly be addressed through a traditional disarmament approach alone [10]. The need for an integrated wide-ranging set of policies, regulations, and measures has been recognised and acknowledged by the States Parties to the Biological and Toxin Weapons Convention (BTWC), the principal international agreement that prohibits the development, stockpiling, acquisition, and retention of biological weapons. Priority areas of work include review and assessment of scientific and technological developments of relevance to the Convention; strengthening national implementation and enhancing compliance with the provisions of the Convention; enhancing international and national capabilities for preparedness, response, and assistance in case of alleged use of biological weapons or suspicious biological events; and strengthening multilateral cooperation and assistance for promoting the peaceful uses of life sciences [11]. The WHO has also underscored the need for an integrated approach to countering natural, accidental, and deliberate disease and recommended that “considerations for [countering] deliberate releases of biological or chemical agents should be incorporated into existing public health infrastructures, rather than developing separate infrastructures.” [12]

### A web of prevention for biosafety and biosecurity

The concept of a ‘web of prevention’ originated in the early 1990s to refer to the set of complementary and mutually reinforcing policies, regulations and measures that need to be in place to counter the development and use of any form of biological and toxin weapons and guarantee that the life sciences are used only for peaceful purposes [13]. Ensuring that the life sciences are not accidentally or deliberately misused and that naturally occurring disease outbreaks are effectively managed in the twenty-first century requires the development of national implementation approaches that integrate the elements of the international biosafety and international biosecurity regimes. The web of prevention is a useful conceptual tool for understanding the need for a greater interaction between these two regimes. Fostering such an understanding is essential for strengthening the health-security interface at local, national, regional, and international level; for promoting cross-sectorial coordination, communication, and cooperation in case of a bioemergency; and for developing balanced mechanisms for mitigating dual-use risks in the life sciences without stifling innovation.

**Fig. 1:**
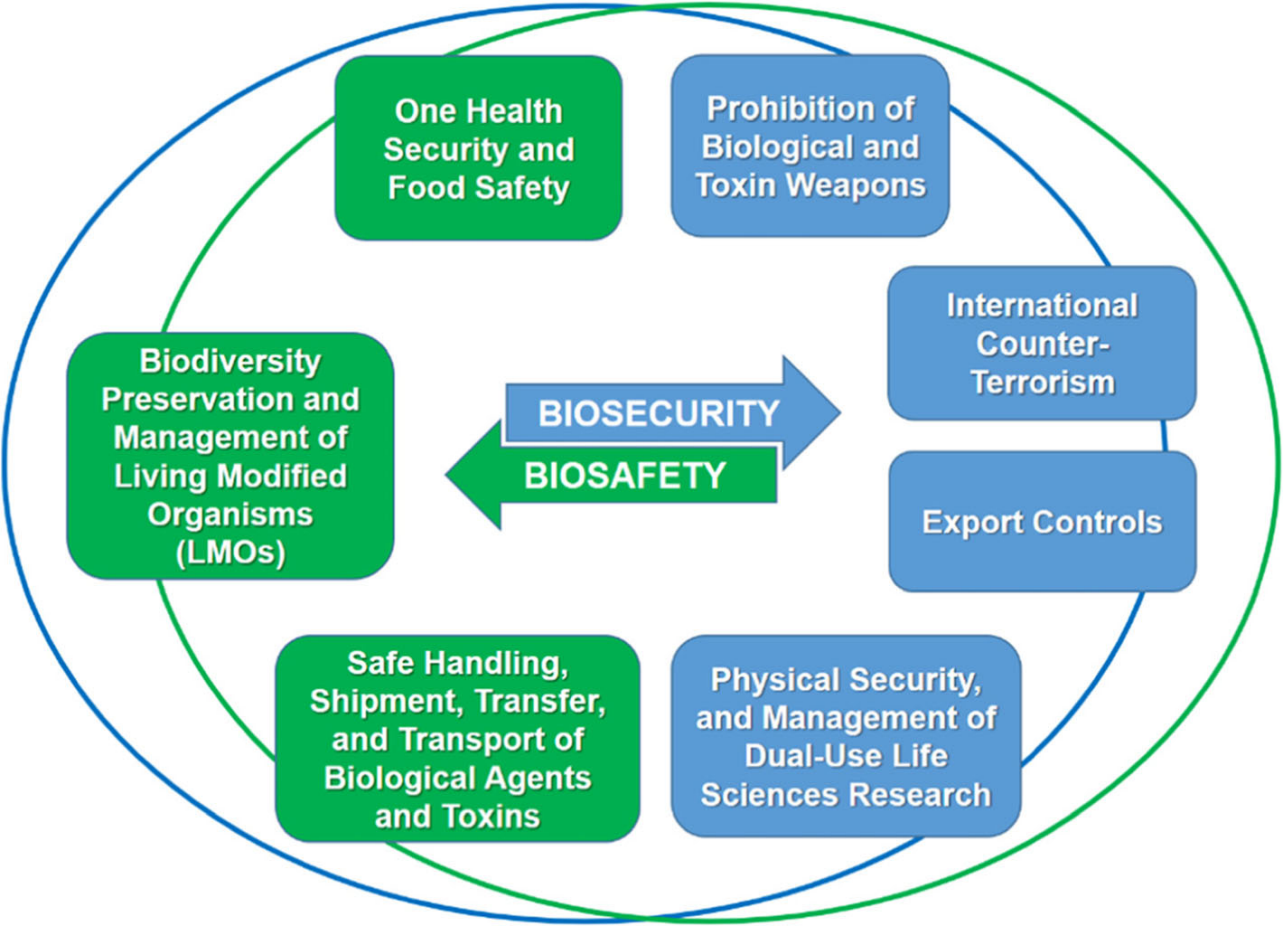
The Web of Prevention for Biosafety and Biosecurity. Source: Authors. The figure has not previously been published. An earlier version of this figure appears in Novossiolova et al. 2019 [14]

The model proposed here (Fig. 1) outlines the main elements of the international biosafety regime and the international biosecurity regime, based on the primary purpose of each of the two regimes: whether it aims to prevent the unintentional (accidental) release of biological agents and toxins, including naturally occurring diseases (biosafety), or whether it aims to prevent the deliberate release of biological agents and toxins (biosecurity).

### International biosecurity regime

The international biosecurity regime seeks to prevent the deliberate misuse of life science knowledge, materials, and technologies in ways that can cause harm to humans, animals, or plants. The regime focuses on upholding the international norm against biological weapons. It comprises four main elements: (1) prohibition of biological and toxin weapons; (2) export controls; (3) international counter-terrorism; and (4) physical security and management of dual-use life sciences research.

#### Prohibition of biological and toxin weapons

The 1975 BTWC prohibits the development, production, stockpiling, acquisition, and retention of ‘microbial or other biological agents, or toxins whatever their origin or method of production, of types and in quantities that have no justification for prophylactic, protective or other peaceful purposes’. [15] A key element of the BTWC is the general purpose criterion which seeks to guarantee a comprehensive international ban on biological and toxin weapons, on the one hand, and ensure the legitimate use of biological agents and toxins for peaceful, prophylactic, and protective purposes, on the other [16]. The state and operation of the Convention is assessed during Review Conferences held every 5 years. The Review Conference is the decision-making body of the Convention. Since 2002, States Parties to the BTWC have agreed an Intersessional Programme of Work between Review Conferences, in order to promote common understanding and effective action on different aspects of the strengthening and implementation of the Convention.

The 1997 Chemical Weapons Convention (CWC) prohibits the development, stockpiling, acquisition, retention, and use of chemical weapons [17]. Both the BTWC and CWC address and prohibit toxins and aim to ensure that advances in biological and chemical sciences are used only for peaceful purposes. The need for sustained engagement between the BTWC and CWC with regard to the effective management and assessment of scientific and technological developments that are covered by both Conventions has been emphasised by the Scientific Advisory Board and the Advisory Board for Education and Outreach of the Organisation for the Prohibition of Chemical Weapons (OPCW) [18].

The use of biological and toxin weapons in armed conflict is prohibited by the 1925 Geneva Protocol [19]. In the event of a suspected deliberate release of bacteriological (biological) or toxin weapons, any affected State, regardless of whether they are a State Party to the BTWC may request that the UN Secretary General Mechanism for Investigation of Alleged Use of Chemical and Biological Weapons (UNSGM) is triggered. The primary purpose of the Mechanism is to ascertain in an objective and scientific manner whether a violation of the 1925 Geneva Protocol has taken place. The UNSGM grants the UN Secretary General the authority to carry out an investigation including dispatching a fact-finding team to the site/s of the alleged incident/s and to report to all UN Member States [20].

In case of a suspected violation of the BTWC, any State Party to the Convention may lodge a complaint with all possible supporting evidence for consideration by the Security Council of the United Nations [15]. States Parties further undertake to provide assistance to any State Party that has been exposed to danger as a result of a violation of the Convention [15]. However, challenges to the practical operationalisation of these key provisions of the Convention still remain, in part due to the absence of a designated international mechanism for coordinating the response to deliberate biological events [21]. The development of a draft International Bio-Emergency Management Framework for Deliberate Events is a flagship initiative that aims to address some of these challenges [22].

#### International counter-terrorism

Whilst the BTWC does not explicitly refer to the use of biological weapons by non-State actors, such as terrorist groups, States Parties are obliged to implement the provisions of the Convention domestically and enforce comprehensive prohibition of biological and toxin weapons in any territory that is under their jurisdiction or control.

United Nations Security Council Resolution 1373 adopted in 2001 stresses the role of international cooperation and data sharing in countering the threat posed by the possession of weapons of mass destruction by terrorist groups [23]. United Nations Security Council Resolution (UNSCR) 1540 adopted in 2004 specifically addresses the risk that non-State actors (defined for the purposes of this resolution as ‘individual or entity, not acting under the lawful authority of any State in conducting activities which come within the scope of this resolution’) ‘may acquire, develop, traffic in or use nuclear, chemical and biological weapons and their means of delivery’. [24] UNSCR 1540 is binding on all States and seeks to ensure the effective implementation, application, and enforcement of domestic controls on the production, use, storage, and transport of biological materials, in order to deter, prevent, detect, and combat their diversion for illicit purposes [24].

The United Nations Office of Counter-Terrorism which coordinates the all-of-UN approach to prevent and counter terrorism and violent extremism aims to promote interagency operability and coordinated communication in case of chemical and/or biological attacks [25].

#### Export controls

The BTWC under Article III prohibits the provision of any form of assistance, including the transfer of biological materials and equipment that may facilitate the development of biological or toxin weapons. UNSC Resolution 1540 further requires that all States should refrain from providing any form of support to non-State actors that attempt to develop, acquire, manufacture, possess, transport, transfer or use nuclear, chemical or biological weapons and their means of delivery [24]. In order to ensure that international exports do not contribute to the development of biological and toxin weapons and meet their obligations under the biological prohibition regime, States need to develop and have in place domestic import and export controls. An example of an ad-hoc international export control arrangement is the Australia Group which was established in 1985 and currently has 43 members and one adherent [26]. Australia Group participants require licences for the export of specific dual-use biological equipment and related technology and software; human and animal pathogens and toxins; and plant pathogens [27].

The Green Customs Initiative, whilst not an export control regime, aims to enhance the capacity of customs and other relevant enforcement personnel to monitor, detect and prevent illicit trade in and trafficking of environmentally-sensitive commodities, including toxic chemical products and toxins, hazardous wastes, endangered species, and living-modified organisms (LMOs) [28]. The Cartagena Protocol on Biosafety to the Convention on Biological Diversity (CBD) establishes a framework for preventing and penalising the illegal transboundary movements of LMOs [29]. To facilitate and ensure the effective implementation of this framework, the Network of Laboratories for the Detection and Identification of LMOs under the Cartagena Protocol is developing a draft training manual which specifically covers considerations for the development of relevant national strategies, including the use of new technology advances that could be applied for detecting LMOs [30].

#### Physical security and Management of Dual-use Life Sciences Research

When considering the national implementation of the Convention, the Eighth Review Conference of the BTWC has recognised the need for life science stakeholder engagement with biological security and noted the value of domestic measures to:

- implement voluntary management standards on biosafety and biosecurity;
- encourage the consideration of development of appropriate arrangements to promote awareness among relevant professionals in the private and public sectors and throughout relevant scientific and administrative activities;
- promote amongst those working in the biological sciences awareness of the obligations of States Parties under the Convention, as well as relevant national legislation and guidelines;
- promote the development of training and education programmes for those granted access to biological agents and toxins relevant to the Convention and for those with the knowledge or capacity to modify such agents and toxins;
- encourage the promotion of a culture of responsibility amongst relevant national professionals and the voluntary development, adoption and promulgation of codes of conduct; [31]

Likewise, the value of engaging science stakeholders in government, industry, and academia with the provisions of the chemical prohibition regime has been recognised by the States Parties to the CWC. The OPCW has published the Hague Ethical Guidelines which are intended to serve as elements for the development of codes of conduct and for raising awareness of the objectives of the Convention among those working in chemistry and related fields [32]. The OPCW Advisory Board on Education and Outreach seeks to provide advice on the development of strategies and key messages for education and outreach activities that support the implementation of the Convention [33].

Both the World Health Organisation (WHO) and the World Organisation for Animal Health (OIE) have sought to engage life science stakeholders with the need for countering the deliberate misuse of the life sciences [34]. Laboratory biosecurity refers to the principles, technologies, and practices that need to be in place to prevent the unauthorised access, loss, theft, misuse, diversion or release of pathogenic biological agents or toxins [35]. Attention is also given to the management of dual-use life sciences research of concern – “life sciences research that, based on current understanding, has the potential to provide knowledge, information, products or technologies that could be directly misapplied to create a significant threat with potential consequences to public health and safety, agricultural species and other plants, animals, and the environment.” [36] Despite its broad scope, this definition provides a useful framework for engaging the life science community with the security implications of novel scientific and technological advances. In this spirit, a recent guidance document of WHO has recommended that national biosecurity frameworks promote regular and comprehensive assessment of the risks related to the hostile misuse of life sciences advances [35]. The OIE has published *Guidelines for Responsible Conduct in Veterinary Research: Identifying, Assessing, and Managing Dual Use* which seek to raise awareness about the dual-use potential of research in veterinary settings – the fact that benignly intended and legitimate life science research could be misused to cause harm to humans, animals, or the environment [37]. The document stresses that the responsibility for the identification, assessment and management of dual-use implications rests to differing degrees across many stakeholders throughout the research life cycle including researchers and their host institutions, grant and contract funders, companies, educators, scientific publishers and other communicators of research, and regulatory authorities [37].

To facilitate engagement among life science stakeholders with issues of dual use and responsible science, WHO has held a series of dialogue interactions with science academies and councils [38], editors and publishers [39], and funders [40]. More recently, WHO has initiated international consultations for the development of a global guidance framework for the governance of dual-use issues in the life sciences [41]. To maximise the impact of this initiative, it is important that any resultant global framework for dual-use risk management is underpinned by a cross-sectorial approach that takes into account the risk of deliberate disease against human, animals, or plants.

### International biosafety regime

The international biosafety regime that seeks to prevent the unintentional release of pathogens and toxins, including naturally occurring diseases comprises three main elements: one health security and food safety; biodiversity preservation and management of living modified organisms (LMOs); and safe handling, including shipment, transport, and transfer of biological agents and toxins.

#### One health security and food safety

The ‘One Health’ concept holds that ‘human health and animal health are interdependent and bound to the health of the [natural] ecosystems in which they exist’. [42] At international level, protecting and advancing human, animal, and plant health falls within the remit of several organisations, including the WHO, OIE, and the Food and Agriculture Organisation of the United Nations (FAO) which also administers the International Plant Protection Convention (IPPC) [43]. There has been a growing recognition that securing global one health requires fostering capacities for the effective prevention, detection, preparedness, and response to disease regardless of its origins. This includes the risk of deliberate biological threats. Under the 2005 International Health Regulations, States are obliged to "develop, strengthen and maintain [ …] the capacity to detect, assess, notify, and report" biological events, as well as to “develop, strengthen, and maintain [ …] the capacity to respond promptly and effectively to public health risks and public health emergencies of international concern.” [44] In 2011, the World Health Assembly of WHO adopted a resolution urging all Member States to:

“to integrate all-hazards health emergency and disaster risk-management programmes (including disaster risk-reduction) into national or subnational health plans and institutionalize capacities for coordinated health and multisectoral action to assess risks, proactively reduce risks, and prepare for, respond to, and recover from, emergencies, disasters and other crises;” [45].

In 2015, the OIE published *Biological Threat Reduction Strategy: Strengthening Global Biological Security* which seeks to develop a sustainable and effective protection against threats from deliberate and accidental releases of animal, including zoonotic pathogens by strengthening existing systems for surveillance, early detection, and rapid response and fostering scientific networks for promoting biosafety and biosecurity [46]. To sensitise veterinary services to the challenges of establishing the origins of a disease outbreak, the OIE has developed a guiding document on the investigation of suspicious biological events in relation to animal health [47]. The OIE has also published guidelines for simulation exercises that could be used to facilitate training on the role of cross-sectorial collaboration in case of bioemergencies [48].

The *FAO Biosecurity Toolkit* that was published in 2007 promotes an integrated approach to the management of biological risks related to food safety, zoonoses, the introduction of animal and plant diseases and pests, the introduction and release of LMOs and their products, and the introduction and management of invasive alien species [49]. The Toolkit seeks to strengthen the capacity of relevant organisations in the area of food safety, public health, agriculture, forestry, fisheries, and environmental protection for effective cross-sectorial coordination and cooperation in case of biological events regardless of their origins.

#### Biodiversity preservation and Management of Living Modified Organisms (LMOs)

Biodiversity plays a critical role in ensuring the health of natural ecosystems and its preservation is indispensable to the implementation of a ‘one health’ approach for realising health security. Given the lasting impact that novel life sciences advances can have on natural ecosystems, it is essential that the application of such advances is carried out in ways that ensure environmental integrity and protect human health. The 1993 Convention on Biological Diversity (CBD) acknowledges the adverse effects that LMOs may have on the conservation and sustainable use of biological diversity and its 2003 Cartagena Protocol on Biosafety sets out specific provisions for assessing and managing the risks related to handling, transfer, and use of LMOs [50]. The *Guidance on Risk Assessment of Living Modified Organisms and Monitoring in the Context of Risk Assessment* developed within the framework of the Cartagena Protocol defines a five-step roadmap for conducting a risk assessment of LMOs which takes into account risks to human health [51]. The Guidance specifically addresses the use of gene-drive systems in relation to living modified mosquito species.

In line with its mandate to monitor and assess the impact of novel biotechnology advances, the CBD Subsidiary Body on Scientific, Technical and Technological Advice (SBSTTA) has recommended the establishment of an Ad Hoc Technical Expert Group on Synthetic Biology which seeks to contribute to the harmonisation of the regulation of organisms, components, or products derived from synthetic biology and identify risks and benefits of the use of synthetic biology techniques to the conservation and sustainable use of biodiversity and related human health and socioeconomic impacts [52]. Key issues that the Group considers include synthetic biology organisms that may fall outside the definition of LMOs and the potential impacts of applications of synthetic biology, including those applications that involve organisms containing engineered gene drives [53].

#### Safe handling, shipment, transport, and transfer of biological agents and toxins

Safety procedures and practices for handling, shipping, transfer, and transport of biological agents and toxins are intended to prevent laboratory acquired infections and ensure that such materials are not accidentally released in the environment. The International Organisation for Standardisation (ISO) has recently published an ISO Management System Standard titled ISO 35001 on Biorisk Management for Laboratories and other Related Organisations [54]. ISO 35001 is intended as a performance-based standard which defines a process to identify, assess, control, and monitor the risks associated with hazardous biological materials [55]. It has been noted that the effective implementation of ISO 35001 by any organisation that works with, stores, transports, and/or disposes of hazardous biological materials can play an important role in achieving the BTWC objectives to enhance the physical security of biological agents and toxins and prevent unauthorised access [55].

Both WHO and OIE respectively have developed a body of guidance on laboratory safety [56]. The 2020 *WHO Laboratory Biosafety Manual – Fourth Edition* adopts a risk- and evidence-based approach to biosafety that emphasises the importance of a comprehensive safety culture in promoting effective and sustainable laboratory practice and risk management [36]. The Manual specifically refers to the need for managing dual-use life sciences research of concern and as such, it seeks to integrate biosafety and biosecurity considerations underscoring the need for assessing and managing the entire scope of biological risks related to life science practice. To this end, the Manual recommends expanding the composition and role of institutional biosafety committees, so that they can be tasked with identifying, assessing, and mitigating potential dual-use risks in the research process (proposal/design stage), during research conduct, and at all communication stages (for example, manuscripts, conferences, presentations) [36].

## Conclusions

As noted in the conclusion of 1997 *JAMA* paper:

“In considering biological control regimes for the future, it is instructive to look back over the developments in both security and environmental controls.” [1]

Strengthening the international systems for countering disease threats regardless of their origins and ensuring that effective mechanisms are in place to prevent the hostile misuse of life sciences advances requires the sustained interaction of biosafety and biosecurity regimes. The web of prevention constitutes a useful conceptual tool for developing an understanding of the complementary relationship of these two sets of instruments and devising integrated policy strategies for promoting the effective national implementation of their provisions. Attention needs to be given to the following steps and measures for strengthening the implementation of all elements of the web of prevention:

### Universalisation of international legally binding instruments

An essential element in the process of harmonising the international biosafety and biosecurity regimes is ensuring that States recognise and respect their legally binding obligations. As of 2021, key international biosafety (e.g. Cartagena Protocol on Biosafety) and biosecurity (e.g. BTWC, CWC, Geneva Protocol) instruments have not been ratified by all States. By joining relevant international agreements, States develop an appreciation of the complementary role of these agreements and are committed to take effective steps for their national implementation. At the same time, international legal frameworks provide a normative and procedural context for dealing with instances of non-compliance. As such, they help reassert the importance of international rules by drawing attention to the reputational, economic, and political costs that States could incur if they fail to abide by these rules.

### Enhancing the interaction between the existing international mechanisms for multilateral negotiations with relevance to biosafety and biosecurity

It is important that the outcomes achieved in one area of biological risk management are shared promptly into the proceedings of other international agreements, in order to ensure cross-fertilisation and effective data sharing. For instance, those working in the field of disarmament need to be kept up-to-date with international policy developments in the field of health, responsible laboratory practice, and LMOs risk assessment, and vice versa.

### Creating platforms for promoting effective action in support of the web of prevention for biological risk management

Biological risk management is complex and multifaceted, and hence it is important that the existing processes for international multilateral negotiations are supplemented with opportunities for a broad stakeholder engagement. Semi-formal or informal forums and platforms (e.g. Global Health Security Agenda, Global Partnership against the Spread of Weapons and Materials of Mass Destruction, EU CBRN Centres of Excellence Initiative) can bring together both government and civil society representatives, including industry and academia, in order to foster the development of innovative solutions and facilitate the transfer of best practices and lessons learned [57]. As such, they can play an instrumental role in informing States’ efforts to promote common understanding and effective action on strengthening the norms of biosafety and biosecurity worldwide.

### Developing integrated approaches for the national implementation of all elements of the web of prevention for biological risk management

International law defines the legal responsibilities of States in their conduct with each other, and their treatment of individuals within State boundaries [58]. With regard to biological risk management, States are required to take all necessary steps to ensure that all elements of the web of prevention are effectively implemented nationally within the territories under their jurisdiction. The implementation of integrated approaches and initiatives that are underpinned by cross-sectorial collaboration at the national level is to be encouraged, in order to ensure that all those engaged in the life sciences whether in government, academia, industry, or as individuals are aware of their responsibility to cause no harm [59].

## Data Availability

Not applicable.

## Abbreviations

BTWC: Biological and Toxin Weapons Convention
CBD: Convention on Biological Diversity
CWC: Chemical Weapons Convention
FAO: Food and Agriculture Organisation of the United Nations
GMOs: Genetically modified organisms
IPPC: International Plant Protection Convention
ISO: International Organisation for Standardisation
JAMA: Journal of the American Medical Association
LMOs: Living modified organisms
OIE: World Organisation for Animal Health
OPCW: Organisation for the Prohibition of Chemical Weapons
SBSTTA: Subsidiary Body on Scientific, Technical and Technological Advice
UNSCR 1540: United Nations Security Council Resolution 1540
UNSGM: UN Secretary General Mechanism for Investigation of Alleged Use of Chemical and Biological Weapons
WHO: World Health Organisation
